# An Outbreak of Chikungunya in Rural Bangladesh, 2011

**DOI:** 10.1371/journal.pntd.0003907

**Published:** 2015-07-10

**Authors:** Selina Khatun, Apurba Chakraborty, Mahmudur Rahman, Nuzhat Nasreen Banu, Mohammad Mostafizur Rahman, S. M. Murshid Hasan, Stephen P. Luby, Emily S. Gurley

**Affiliations:** 1 Epidemiology Department, Institute of Epidemiology Disease Control and Research, Dhaka, Bangladesh; 2 Institute of Epidemiology Disease Control and Research, Dhaka, Bangladesh; 3 Department of Outbreak and Surveillance Research, Centre for Communicable Diseases, International Centre for Diarrhoeal Disease Research (icddr,b), Dhaka, Bangladesh; 4 Department of Infectious Diseases, Stanford University, Stanford, California, United States of America; Florida Department of Health, UNITED STATES

## Abstract

**Background:**

The first identified Chikungunya outbreak occurred in Bangladesh in 2008. In late October 2011, a local health official from Dohar Sub-district, Dhaka District, reported an outbreak of undiagnosed fever and joint pain. We investigated the outbreak to confirm the etiology, describe the clinical presentation, and identify associated vectors.

**Methodology:**

During November 2–21, 2011, we conducted house-to-house surveys to identify suspected cases, defined as any inhabitant of Char Kushai village with fever followed by joint pain in the extremities with onset since August 15, 2011. We collected blood specimens and clinical histories from self-selected suspected cases using a structured questionnaire. Blood samples were tested for IgM antibodies against Chikungunya virus. The village was divided into nine segments and we collected mosquito larvae from water containers in seven randomly selected houses in each segment. We calculated the Breteau index for the village and identified the mosquito species.

**Results:**

The attack rate was 29% (1105/3840) and 29% of households surveyed had at least one suspected case: 15% had ≥3. The attack rate was 38% (606/1589) in adult women and 25% in adult men (320/1287). Among the 1105 suspected case-patients, 245 self-selected for testing and 80% of those (196/245) had IgM antibodies. In addition to fever and joint pain, 76% (148/196) of confirmed cases had rash and 38%(75/196) had long-lasting joint pain. The village Breteau index was 35 per 100 and 89%(449/504) of hatched mosquitoes were *Aedes albopictus*.

**Conclusion:**

The evidence suggests that this outbreak was due to Chikungunya. The high attack rate suggests that the infection was new to this area, and the increased risk among adult women suggests that risk of transmission may have been higher around households. Chikungunya is an emerging infection in Bangladesh and current surveillance and prevention strategies are insufficient to mount an effective public health response.

## Author Summary

Chikungunya virus is transmitted through bites from *Aedes* mosquitoes and causes outbreaks of fever and polyarthralgia; the geographic range of infection is expanding. An outbreak of fever with prolonged joint pain was investigated in Bangladesh in 2011, where house-to-house surveys were carried out to identify suspected cases. Twenty-nine percent of the village inhabitants experienced symptoms consistent with Chikungunya during the three months of the outbreak. Eighty percent of suspected cases had evidence of IgM antibodies against Chikungunya suggesting that this virus caused the outbreak. Attack rates were similar for all age groups, which suggests that this population had little pre-existing immunity to the disease. This is consistent with the assumption that Chikungunya is an emerging infection in this part of the world where the majority of people likely remain susceptible to infection. Attack rates were higher among adult females, which may provide clues to where transmission occurs. Since most rural women spend the majority of their time in and around the home, interrupting vector habitat near houses might be a useful way to control epidemics. Given the continued risk for outbreaks, we need more efficient methods for detection and control.

## Introduction

Chikungunya is an arthropod-borne disease caused by Chikungunya virus (Alphavirus family, Togaviridae family) which was initially identified in Tanzania in 1952 [1]. Chikungunya outbreaks likely happened before the virus was identified because there were many verifiable depictions of epidemic fevers with remarkable arthralgia [2]. Humans can be a reservoir for Chikungunya virus during epidemics. In the past 50 years, Chikungunya has re-emerged in several occasions in both Africa and Asia [3]. Rapid and local transmission of Chikungunya occurred in the Caribbean and the Americas within 9 months during 2013–2014 [4].*Aedes* mosquitoes transmit Chikungunya virus. *Aedes aegypti mosquitoes* are responsible for transmission of both Chikungunya and dengue [5]and in Asia, have been identified as the primary vector in most urban dengue epidemics [6].*Aedes albopictus* was identified as the vector in the 2006 Chikungunya outbreak in La Reunion (an island in the Indian Ocean). This newly identified vector caused effective replication and spread the infection beyond previously endemic areas [6].*A*.*albopictus* can prosper in both rural and urban environments [7] and breed in artificial water containers [8].

Since 2005, Chikungunya has become an emerging public health problem in Southeast Asia, with large numbers of cases reported in Singapore, Malaysia, and Thailand [9]. In 2006, an increase in the incidence of Chikungunya in India prompted testing of serum samples collected from febrile patients from two different surveillance projects in Dhaka, Bangladesh. One hundred seventy-five serum samples were tested however none had antibodies against Chikungunya virus [10]. In 2008, the first recognized outbreak of Chikungunya in Bangladesh was identified in the northwest area of the country. Transmission appeared to be geographically limited to two villages bordering India in northwestern Bangladesh [11].

In late October 2011, an outbreak of fever and severe joint pain was reported by a local health official in Dohar Sub-district in Dhaka District. Limited antibody testing for dengue and blood smears for malaria conducted at the local health clinic suggested that the illnesses were not caused by dengue or malaria. On November 2, 2011, an outbreak investigation team comprised of medical epidemiologists, entomologists, field research assistants and laboratory technicians from the Institute of Epidemiology Disease Control and Research (IEDCR), of the Bangladesh Ministry of Health and Family Welfare, and icddr,b (formerly known as the International Centre for Diarrhoeal Disease Research, Bangladesh) began an investigation with the objectives of identifying the etiology of the outbreak, describing the clinical presentation of cases, and identifying associated vectors.

## Methods

We focused the investigation in Char Kushai village because a review of the log books from the local public hospital showed that 70% of the inpatients and outpatients who sought care for fever and joint pain during May—October 2011 were from that village. We conducted house-to-house surveys to identify and enlist suspected cases, defined as any inhabitant of Char Kushai village who reported fever followed by joint pain in the extremities with onset since August 15, 2011. Local authorities reported that the outbreak had been ongoing since May, but we limited our suspected case finding efforts to those occurring since August due to concerns about the ability of residents to reliably recall illnesses for more than a few months.

Patients meeting the suspected case definition were asked to visit the local health clinic to provide a blood specimen for laboratory testing. Patients who came to the clinic were also interviewed by the investigation team about their socio-demographic, clinical and travel histories using a structured questionnaire. Blood was tested in the IEDCR laboratory for IgM antibodies to Chikungunya virus using an antibody enzyme-linked immunosorbent assay (ELISA) test kit (Bioline Chikungunya IgM manufactured by Standard Diagnostics Inc., Yongin-si, South Korea). Confirmed cases were defined as suspected cases with IgM antibodies against Chikungunya. Acute serum collected from patients within 2 days of symptom onset were tested for using quantitative reverse transcription polymerase chain reaction (RT-qPCR) to identify Chikungunya virus RNA [12]. We used suspected cases to estimate attack rates by age and gender and described the clinical characteristics of laboratory confirmed cases.

IgM antibodies against Chikungunya typically start to be detected 7 days post-onset of illness and by two weeks post-onset, nearly all patients will have detectable IgM antibodies. These persist for approximately 2 months before IgM begins to decline [13–15]. We calculated the total proportion of patients tested that had IgM antibodies and also for each of these categories of time since illness onset.

For the entomological investigation, the village was divided into nine segments of approximately equal areas. Seven households were randomly selected in each segment using household line listings from the house-to-house survey and larvae were collected during November 2–21 following the World Health Organization’s guidelines for vector surveillance [8]. The team inspected all water containers in and surrounding each selected house, recorded whether larvae were observed, and collected larvae if present. Larvae were hatched and identified at the IEDCR entomology lab. The Breteau index (number of positive containers per 100 houses inspected) was calculated for the village to estimate the mosquito population density in the area [16].

### Ethics

This investigation was carried out as a response to an outbreak investigation and thus the protocol was not reviewed by a human subjects committee. However, participants provided verbal informed consent prior to interviews and blood specimen collection and the Government of Bangladesh approved the outbreak investigation plan.

## Results

Data collectors surveyed all 897 households in the village and collected information regarding symptoms for all 3,840 residents; 1105 (29%) of household members met the suspected case definition. There were no differences in attack rates by gender among children <10 years of age; however, females were more likely to report illness than males for every other age group and the differences were greatest among residents aged 31–40 years (28% of males vs 50% of females) and 41–50 years (29% vs 53%) (Table 1). Sixty-four percent of households had at least one suspected case, while 15% had three or more (Table 2).

**Table 1:** Attack rates of suspected Chikungunya fever by age and gender in Char Kushai, Dohar, Bangladesh, August 15–2 November 2011.

Age groups	Gender	
	Male	Female
<10 years	93/496 (19%)	86/463 (19%)
10–20 years	72/386 (19%)	102/395 (26%)
21–30 years	52/244 (21%)	139/417 (33%)
31–40 years	56/203 (28%)	157/316 (50%)
41–50 years	53/185 (29%)	114/215 (53%)
51–60 years	46/131 (35%)	50/125 (40%)
> 60 years	41/138 (30%)	44/126 (35%)
Total	413/1783(23%)	692/2057(34%)

**Table 2:** Number of suspected cases per household (N = 897) in Char Kushai, Dohar, Bangladesh, August 15–2 November 2011.

No. of suspected cases	Households, n(%)
0	323 (36)
1	263 (29)
2	178 (20)
≥3	133 (15)

Twenty-two percent of suspected cases (245/1105) provided blood for testing and 80% (196/245) of them had IgM antibodies against Chikungunya virus. Suspected cases that selected for testing were similar in age to those who were not tested, with the exception that fewer children aged <10 presented for a blood draw. In addition, cases who sought testing were more likely to be female than suspected cases who were not tested (Table 3). Patients tested within 1 week of illness onset were unlikely to have IgM antibodies, while 93% of suspected cases tested between 30 and 60 days post illness onset had IgM antibodies against Chikungunya (Table 4). Confirmed cases had dates of illness onset from August through November, per the suspected case definition. One case patient provided a blood sample within 2 days of onset and this patient had detectable Chikungunya virus RNA with RT-qPCR; this patient did not have IgM antibodies in serum.

**Table 3:** Comparing the age and sex of suspected cases who presented to the clinic for antibody testing with suspected cases who were not tested, Char Kushai, Bangladesh, 2011.

	Suspected cases tested, N = 245	Suspected cases not tested, N = 860
	n(%)	n(%)
Age groups (in years)		
<10	23 (9)	156 (18)
10–20	33 (14)	141 (16)
21–30	39 (16)	152 (18)
31–40	54 (22)	159 (19)
41–50	47 (19)	120 (14)
51–60	27 (11)	69 (8)
>60	22 (9)	63 (7)
Female	177 (72)	514 (60)

**Table 4:** Proportion of suspected patients tested with evidence of IgM antibodies against Chikungunya virus in serum by days since illness onset, Char Kushai, Bangladesh 2011, N = 245.

Days since illness onset	No. and proportion positive
<7	1 (9)
7–15	16 (64)
15–30	33 (72)
30–60	63 (93)
>60	83 (87)
Total	196 (80)

Thirty-eight percent (75/196) of laboratory confirmed cases reported having joint pain that persisted for more than one month and 76% (148/196) reported a rash (Table 5). Most rashes were macular (77%) and involved the face (67%) and upper extremities (53%). Among cases reporting joint pain, the median number of joints affected was nine (range: 1–14). The most commonly affected joint was the knee (36%). Joint pain was accompanied by swelling in 29% of the cases which subsided with the remission of joint pain.

**Table 5:** Clinical symptoms of patients who self-selected for laboratory testing and had IgM antibodies against Chikungunya virus in serum in Char Kushai, Dohar, Bangladesh, 2011 (N = 196).

Symptoms	n (%)
Fever	196 (100)
Joint pain	196 (100)
Rash	148 (76)
Itching	97 (50)
Joint pain lasting>1 month	75 (38)
Joint swelling	56(29)
Headache	23 (12)
Weakness	12 (6)

Sixty-three houses were inspected for water containers with mosquito larvae, and 252 artificial **and natural** containers with water were found and 534 larvae were collected. Eighty-nine percent of larvae (449/504) that hatched yielded *A*. *albopictus* mosquitoes and the remaining yielded *Culex quinquefaciatus* mosquitoes. No *A*. *aegypti* mosquitoes hatched. The overall Breteau index for the village was 35 per 100.

## Discussion

Laboratory findings confirmed that Chikungunya virus caused this outbreak and the clinical features were consistent with previously described outbreaks [17, 18]. This investigation provides further evidence that Chikungunya virus has become an emerging public health problem in Bangladesh [11]. Though no recent community seroprevalence studies of Chikungunya have been published from Bangladesh or nearby countries, a 1995 cross-sectional survey carried out in Kolkata, which is approximately 250 km from Dhaka, indicated that the level of previous exposure to Chikungunya infection in that city was low [19]. Chikungunya infection gives life-long immunity [20], so the consistently high attack rates by age group in our investigation suggest that Chikungunya was new to this geographic area. An abundance of a particular species of mosquitoes during an outbreak is an important condition for determining the vector responsible for transmission [21] and the fact that *A*.*albopictus* hatched from 89% of the larvae collected in the village suggests that this vector was likely responsible for transmission during this outbreak. As *A*. *albopictus* has a tendency to breed in water compartments close to homes and to feed during the day [22], persons who are at home during the day time could be at increased risk due to prolonged exposure to these mosquitoes. Adult women, most of whom spend the majority of their day at or very near the home, experienced the highest attack rates in this outbreak. This finding is similar to outbreaks of Chikungunya in rural areas in other countries where higher risk among women was also reported [23, 24].

According to WHO, places with a Breteau index >20% have a high risk for dengue outbreaks [16], and this may be true for other outbreaks of mosquito-borne illness as well. In this outbreak, the Breteau index was 35%, suggesting risk of transmission of mosquito-borne disease in Char Kushai was very high. Based on published data on the clinical presentation of Chikungunya, patients’ symptoms usually resolve within a few weeks [25, 26]. However, the joint pain associated with Chikungunya virus infection can persist for weeks or months, and in some cases for years [24, 27], resulting insignificant economic burden due to this disability. In India, the national burden of Chikungunya during the 2006 epidemic was estimated at 25,588 disability adjusted life years (DALYs) lost, with an overall burden of 45.3 DALYs per million (range 0.01 to 265.6 per million in different states); persistent arthralgia accounted for 69% of the total DALYs [28]. In this outbreak, 38% of confirmed Chikungunya cases had joint pain lasting more than one month which increased the burden from the outbreak beyond acute febrile illnesses it caused. However, the patients who were tested and had their clinical features assessed during our investigation self-selected for this additional clinical assessment so were more likely to be severely ill than other suspected cases who did not present for assessment. This self-selection is the best explanation for why their clinical profile is more severe compared to other published reports.

This investigation was subject to several limitations. First, four other villages in the sub-district also reported cases, but we only investigated one village and our findings from Char Kushai may not be representative of all of the affected areas. Second, we aimed to detect symptomatic illness and did not look for asymptomatic infections. Therefore, we likely underestimated the number of people infected during the outbreak given evidence that 3–25% of Chikungunya virus infections are asymptomatic [29, 30]. Third, residents may have been unable to reliably recall their symptoms or onset of illness, particularly for milder illnesses, which may have led to an underestimation of suspected cases. Likewise, some suspected cases may have been missed during the survey. Women, who were almost always interviewed, may have recalled their own illnesses more than illnesses of adult men in the home so this could explain some of the variation in attack rates by gender. However, we would expect that women would be able to recall illnesses among children very well and children were also less likely than women to report systems consistent with the suspected case definition. Therefore, we believe that it is unlikely that the gender differences in attack rates are entirely explained by recall bias.

Resource constraints limited our ability to test all suspected cases for antibodies, and the diagnostic test we used was imperfect, with 84% sensitivity and 91% specificity during the convalescent phase of illness [31]. Therefore, we likely missed some true cases; our analysis showing that patients with recent onset were less likely to have IgM antibodies also suggests that we preferentially missed true cases if they were tested early in their illness. However, our attack rates were similar to other confirmed Chikungunya outbreaks and the symptoms exhibited by confirmed cases were consistent with clinical descriptions of disease from other studies. We did not collect date of onset data from all suspected cases which limits our ability to describe the timing of outbreak peaks. The cases who did present for blood draws and data collection about their illness history self-selected for this data collection and may have been motivated to participate because they had severe illness. Therefore, our clinical description may overestimate the severity of infections during this outbreak. The entomologic survey was conducted at the end of the outbreak; therefore, it is possible that the mosquito species most abundant at the beginning of the outbreak were different than those that we found at the end. However *A*. *albipictus* have been associated in several Chikungunya outbreaks [32], and we found no evidence of *A*. *aegypti* in our survey. It is possible that *A*.*aegypti* played a significant role in transmission at the beginning of outbreak and then disappeared; however, the simplest explanation for our findings is that *A*.*albopictus* were primarily responsible for the outbreak.

This investigation suggests that rural Bangladeshi populations are at risk for emerging mosquito-borne diseases, such as Chikungunya. Efforts to improve surveillance and identify outbreaks more quickly could provide an opportunity for public health action to reduce transmission, such as mosquito control. However, in rural Bangladesh, no public initiatives are currently implemented for mosquito control. WHO guidelines suggest that environmental interventions, such as destroying natural and human-made mosquito breeding sites in and around homes, may be more cost- effective than chemical methods to kill larva and adult mosquitoes [16]. Research to develop and test low-cost methods to identify and respond to outbreaks of mosquito-borne infections in low-income countries should be explored. Ecological studies to better describe the spatial and temporal distribution of vector habitats could help explain why outbreaks in Bangladesh remain geographically limited and could be used to target interventions in populations at the highest risk for vector-borne diseases.

## Supporting Information

S1 Checklist STROBE checklist. (DOC)

S1 Data Data files: a. Chik data. b. HH data. c. HH members information. d. HH susp members. e. Household all.
(ZIP)
